# Multicenter Evaluation of Telehealth Utilization in Hip and Knee Arthroplasty Before and for One Year During the COVID-19 Pandemic

**DOI:** 10.1016/j.artd.2021.09.012

**Published:** 2021-10-02

**Authors:** Stefano Bini, Yu-Fen Chiu, Michael Ast, Chad Krueger, Joseph Maratt, Ilya Bendich

**Affiliations:** a Department of Orthopaedic Surgery, University of California, San Francisco, San Francisco, CA, USA; b Adult Reconstruction and Joint Replacement, Hospital for Special Surgery, New York, NY, USA; c Department of Orthopaedic Surgery, Rothman Institute, Philadelphia, PA, USA; d Department of Orthopaedic Surgery, Methodist Hospital, Indianapolis, IN, USA

**Keywords:** Primary hip replacement, Primary knee replacement, Telehealth, Healthcare delivery, COVID-19

## Abstract

**Background:**

The Coronavirus Disease 2019 (COVID-19) pandemic has led to an increase in telehealth utilization across the health-care sector. It is unknown if telehealth use among hip and knee arthroplasty clinics has remained an important health-care delivery platform. The purpose of the present study was to analyze telehealth utilization before and for 1 year during the pandemic among four varied hip and knee arthroplasty clinics.

**Methods:**

Retrospective data were available from four regionally diverse hip and knee arthroplasty centers. Data on volume of patient visits, demographics, visit types (new visit, follow-up, postoperative visit, other), and visit modality (in-person, telehealth, telephone) were available from January 2020 through April 2021. Data from the centers were analyzed as a total and separately, using chi-squared and Fisher exact tests.

**Results:**

Among the four centers, there were 296,540 hip and knee arthroplasty outpatient clinic visits between January 2020 and April 2021. Of those, 15,240 (5%) were telehealth visits. Before March 2020, less than 0.1% of visits across centers occurred over telehealth. The highest utilization of telehealth visits occurred in March 2020 (>55%) and April 2020 (>25%). From August 2020 until April 2021, telehealth visits accounted for 2%-3% of total visits. Younger patients (<50 years old) were most likely to use telehealth. Follow-up and postoperative were the most likely telehealth visits.

**Conclusion:**

Telehealth utilization peaked during March and April of 2020 and has since reverted to near prepandemic levels. Younger patients and lower complexity visits such as postoperative or follow-up visits are more likely to use telehealth.

## Introduction

The Coronavirus Disease 2019 (COVID-19) pandemic has impacted the World in innumerable ways. In response to the early COVID-19 pandemic, orthopedic surgery departments across the United States (US) used telehealth services to deliver patient care [1]. The implementation of telehealth was further supported by the Centers for Medicare and Medicaid Services. As of March 2020, the Centers for Medicare and Medicaid Services provided payment parity for telehealth services and allowed for a wide variety of communication platforms to deliver telemedicine, whether they were compliant with the Health Insurance Portability and Accountability Act (HIPPA) or not [2].

Telemedicine and telehealth are often interchangeable terms, both referring to the provision of health-care services from provider to patient in differing locations [3,4]. Before COVID-19 being declared a pandemic by the World Health Organization (WHO) on March 11, 2020 [3], telehealth accounted for less than 1% of all patient visits in the US [4]. After the COVID-19 pandemic, however, 83% of academic orthopedic surgery departments implemented telehealth services [1].

Within orthopedic surgery, total joint arthroplasty providers were early adopters of telehealth [[5], [6], [7]]. Although increased utilization of telehealth was evident in the first several months of the COVID-19 pandemic, it is not known if this represents a lasting or transient care delivery model in arthroplasty [7]. The purpose of this study is to analyze the trends of utilization of telehealth among four varied arthroplasty practices across the US from January 2020 to April 2021. Second, we sought to determine if telehealth is more used among certain patient demographics or specific appointment types.

## Material and methods

Data were retrospectively collected from the electronic medical records of four medical centers located in representative areas of the US. Institution review board approval was obtained at each center.

### Patients and centers

The patients included in the study were limited to those evaluated in hip and knee arthroplasty clinics among the four included centers. Demographic data were available. The included centers were anonymized and labeled A, B, C, and D. Center A is an academic, specialized orthopedic hospital in the Eastern region of the US. Center B is an academic medical center in the Western region of the US. Center C is a large community-based private clinic in the Midwestern region of the US. Center D is an academic, specialized orthopedic hospital in the Eastern region of the US.

### Visit types

Visit types encompassed all outpatient visits to an adult hip and knee reconstruction clinic at one of the four centers. Visits were classified as “new patient visits,” “follow-up visits,” “postoperative visits,” and “other visits.” Visits were also characterized by whether they occurred in-person, over telehealth platforms, or over telephone. These data were reported monthly by each site.

### Data compilation and reporting

To provide anonymity to each of the four centers, the total volume of visits in the four centers were reported in absolute values. The data for each center are otherwise reported in percentages rather than absolute values.

### Data analysis

Patient visits to each center, subclassified by sex, appointment types, and age groups, were presented as frequencies and percentages and compared using the chi-squared test or the Fisher exact test, as appropriate. The comparisons for utilization difference in the trends over time between centers were conducted using a Cochran-Armitage test for trend. All tests were 2-sided. Significance was defined as *P* < .05. Statistical analyses and data visualizations were performed using SAS 9.4 (SAS Institute Inc., Cary, NC) and Rstudio 1.2.5042 (RStudio, Inc., Boston, MA).

## Results

### Overall study period results

**Figure 1:**
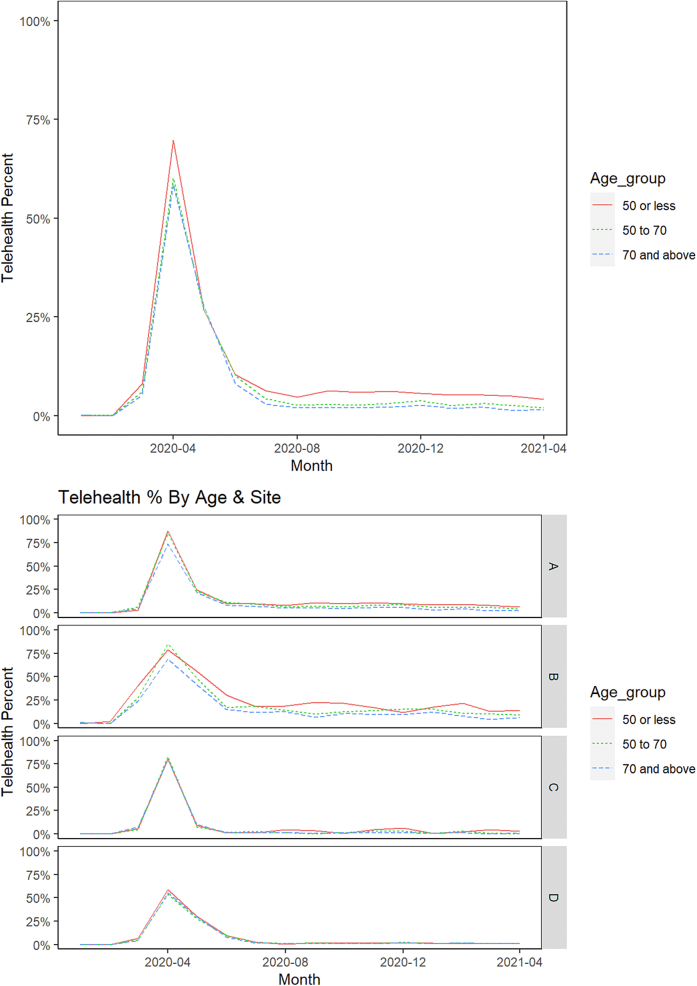
Overall and center-specific breakdown of telehealth usage by age over time.

A total of 296,540 hip and knee arthroplasty outpatient clinic visits occurred between January 2020 and April 2021 among the four centers included in this study. Of these, a total of 15,240 (5%) were telehealth visits. Centers had a difference in overall telehealth utilization during the study period (Table 1). Fifty-seven percent of all visits and 57% of all telehealth visits were by females. Furthermore, during this time period, the highest utilization category for telehealth was among follow-up visits and in patients younger than 50 years (Table 2; Fig. 1).

**Table 1:** Total and center-specific utilization of in-person, telehealth, and telephone visits broken out by sex, appointment type, age group.

Variable	Total N = 296,540)		Center A	Center B	Center C	Center D
	n	%	%	%	%	%
Utilization						
In-person	280,559	95	92	84	96	96
Telehealth	15,240	5	7	14	4	4
Telephone	741	0	1	2	0	0
Sex						
Female	170,296	57	58	61	57	57
Male	126,034	43	42	39	43	43
N/A	210	0	0	0	0	0
Appt						
FollowUp	120,949	41	41	52	1	42
NewPatient	37,623	13	31	29	18	6
Other	93,730	32	5	0	55	40
PostOp	44,236	15	24	19	27	12
Age group						
50 or Less	15,432	5	9	9	19	3
50 to 70	163,175	55	55	54	51	55
70 and Above	117,911	40	36	37	30	41
N/A	22	0	0	0	0	0

Cochran-Armitage test for telehealth utilization comparison between centers						
	AB	AC	AD	BC	BD	CD
*P* value	<.0001	<.0001	<.0001	<.0001	<.0001	0.039

Appt, Appointment type.

Cochran-Armitage test included to assess differences in telehealth-specific utilization between centers.

**Table 2:** Total and center-specific utilization of telehealth visits by sex, appointment type, age group.

Variable	Total (N = 15,240)		Center A	Center B	Center C	Center D
	n	%	%	%	%	%
Sex						
Female	8651	57	58	61	56	55
Male	6580	43	42	38	44	45
N/A	9	0	0	0	0	0
Appt						
FollowUp	9080	60	57	78	5	60
NewPatient	1542	10	17	14	0	6
Other	1532	10	0	0	92	13
PostOp	3084	20	26	8	2	20
Age group						
50 or Less	1140	7	11	13	25	4
50 to 70	8874	58	60	58	49	58
70 and Above	5226	34	28	29	25	39

Appt, Appointment type.

### Telehealth utilization over time

**Figure 2:**
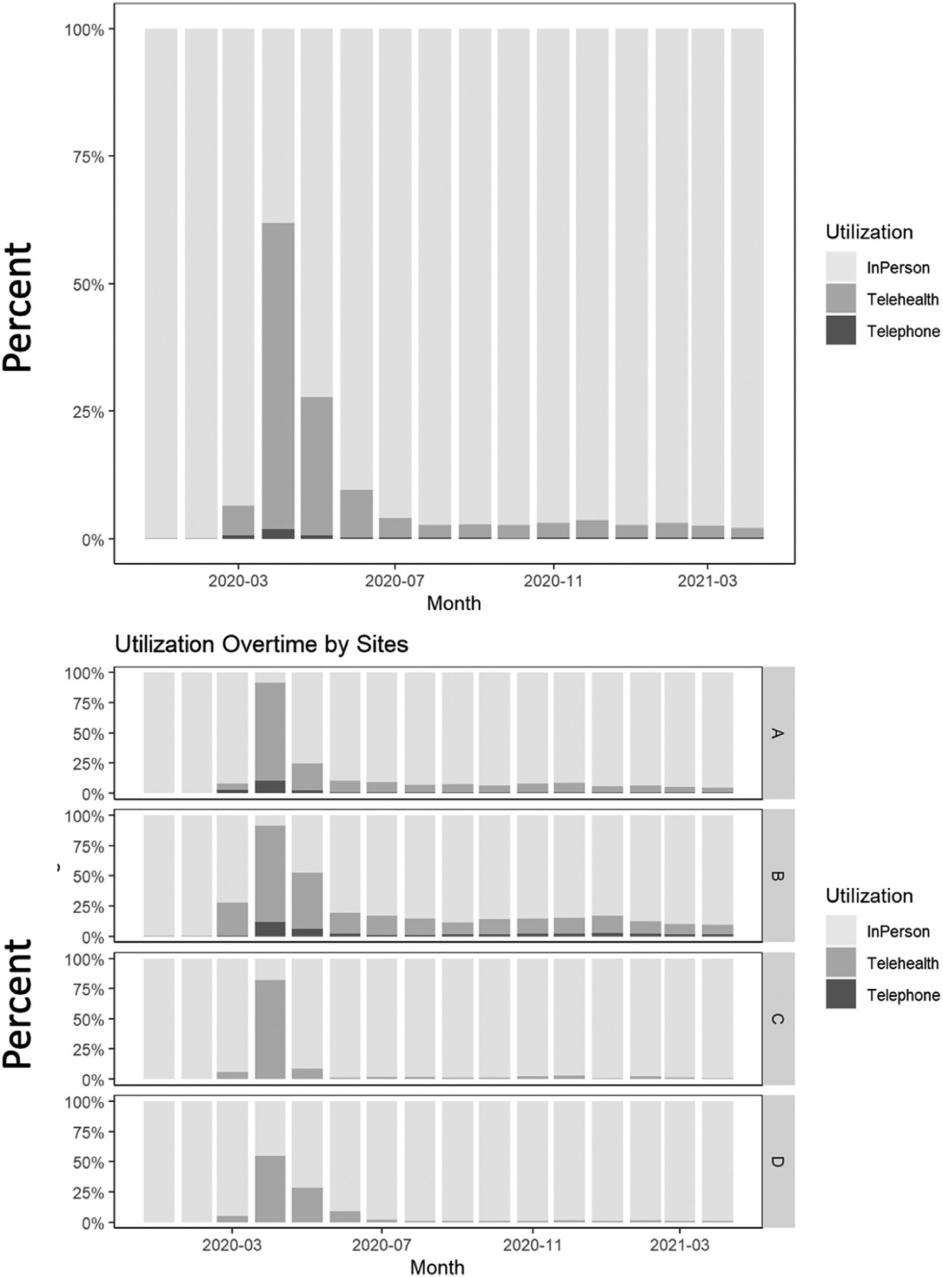
Utilization of of in-person, telehealth, and telephone visits over time (January 1, 2020 – April 30, 2021) for the total cohort and by center.

**Figure 3:**
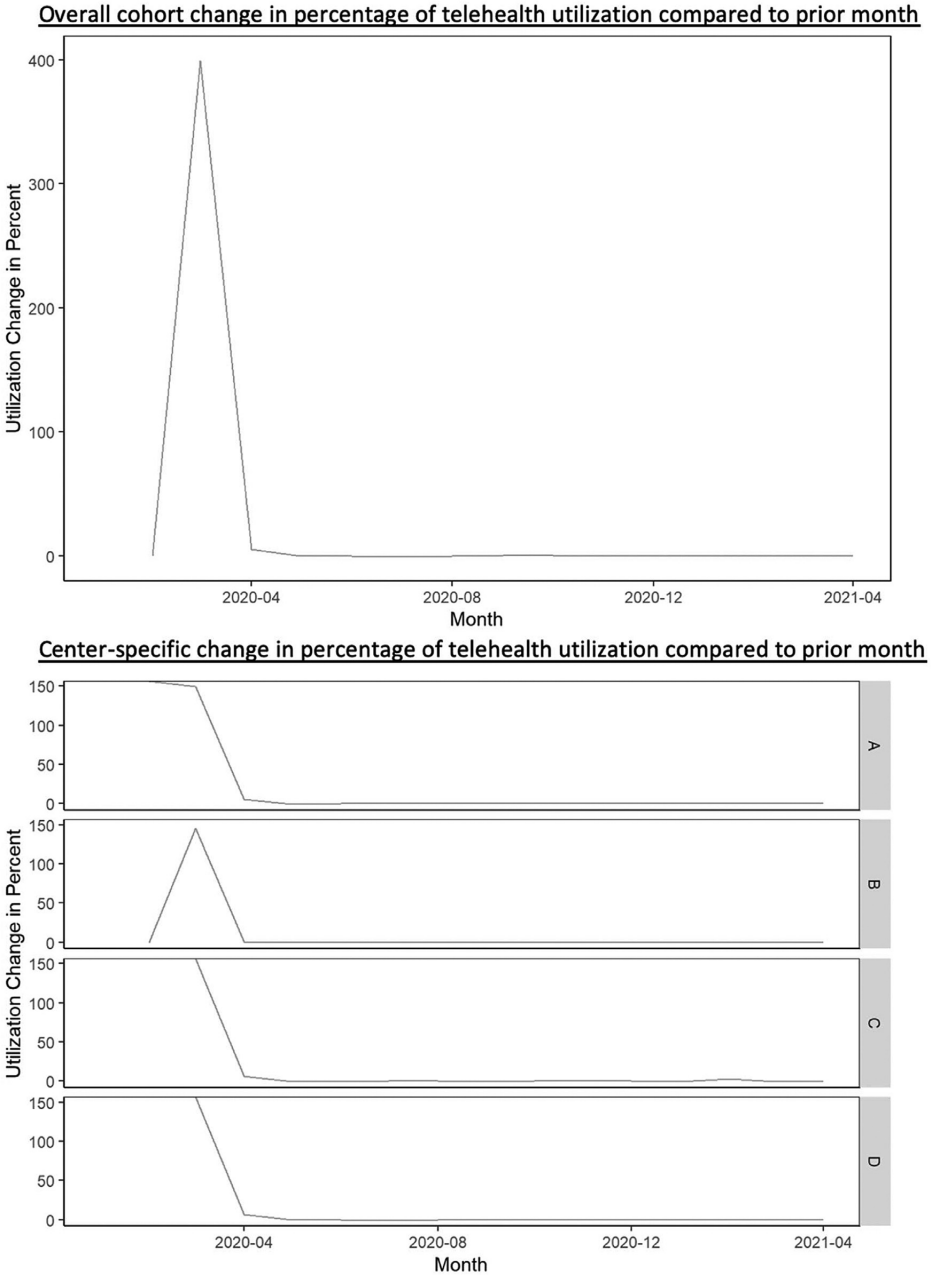
Month-over-month change in telehealth utilization for overall cohort and by center.

Before March 2020, nearly no telehealth visits occurred at any of the centers. The greatest utilization of telehealth visits occurred in April 2020 (center A 82%, center B 79%, center C 82%, center D 55%) across centers (Fig. 2). Utilization of telehealth visits from August 2020 to April 2021 stabilized around 2%-3% of total visits, yet there were significant differences in telehealth utilization trends across centers (Fig. 3; pairwise comparisons between centers, all *P* < .0001, except C-D, *P* = .039)

### Demographics and telehealth utilization

**Figure 4:**
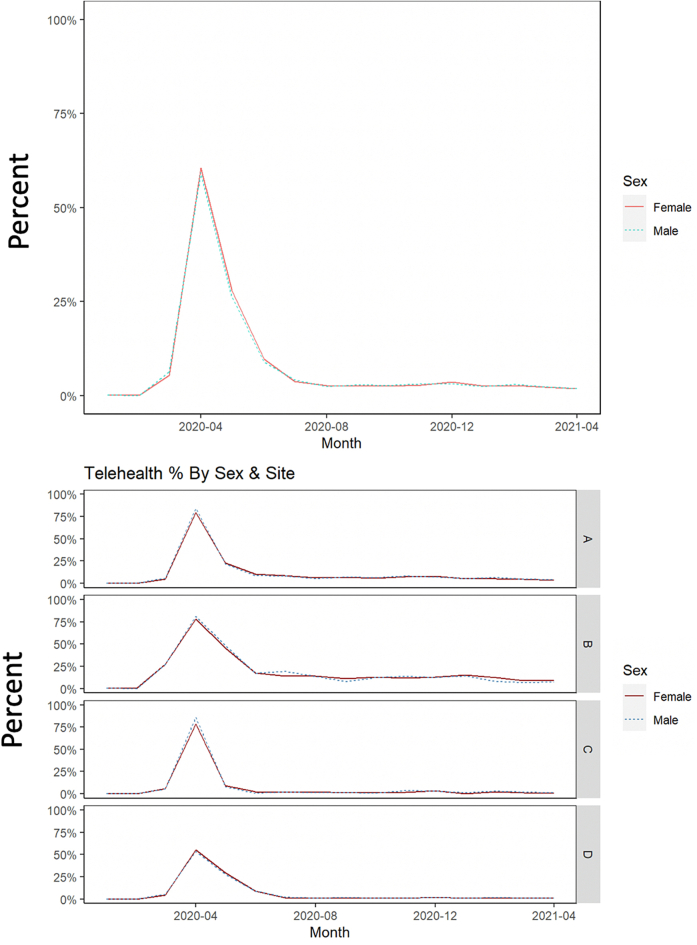
Overall and center-specific breakdown of telehealth usage by sex over time.

There were no differences in telehealth utilization between male and female patients in total and across sites (Fig. 4; April 2020, *P* = .614; August 2020, *P* = .152; December 2020, *P* = .498; April 2021, *P* = .213). Patients younger than 50 years have had the highest utilization of telehealth visits since April 2020. This difference was noted at each center except for center D (Fig. 1).

### Appointment type and telehealth utilization

**Figure 5:**
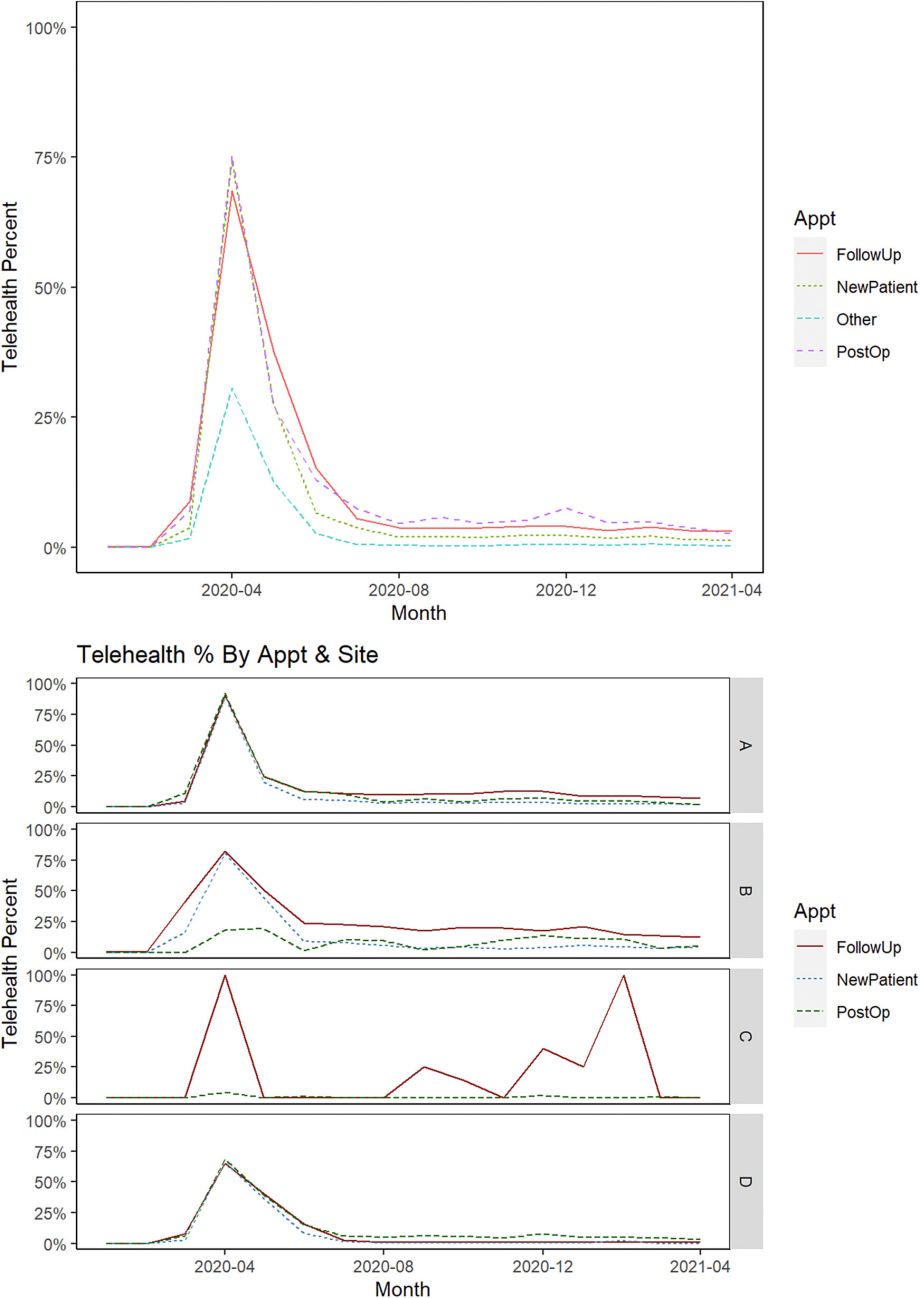
Overall and center-specific breakdown of telehealth usage by appointment type over time.

Across the cohort, from April 2020 to April 2021, telehealth utilization was greatest for follow-up and postoperative visits (Fig. 5). However, centers A, B, C, and D had statistical differences in telehealth utilization by appointment type, with greater utilization among follow-up and postoperative visits in all centers but one (Table 3; *P* < .0001).

**Table 3:** Center-specific breakdown of telehealth usage by appointment type over time (four time periods: 04/2020, 08/2020, 12/2020, 04/2021).

Center A	2020-04	2020-08	2020-12	2021-04	*P* value
	%	%	%	%	
Appt					<.0001
FollowUp	37	67	60	77	
NewPatient	20	15	11	13	
Other	0	0	0	0	
PostOp	43	18	29	10	
Center B					
Appt					<.0001
FollowUp	83	72	67	70	
NewPatient	16	11	7	19	
Other	0	0	0	0	
PostOp	1	18	25	11	
Center C					
Appt					<.0001
FollowUp	7	0	10	0	
NewPatient	0	0	0	0	
Other	93	100	70	100	
PostOp	1	0	20	0	
Center D					
Appt					<.0001
FollowUp	65	36	32	47	
NewPatient	7	2	1	1	
Other	14	6	7	8	
PostOp	15	56	60	44	

Appt, Appointment type.

## Discussion

This multicenter study, which included four regionally diverse centers, analyzed hip and knee arthroplasty clinic telehealth utilization patterns starting from 2 months before the COVID-19 pandemic through 1 year after the start of the pandemic. There were several key findings in this study. First, despite an initial surge in telehealth utilization during the early pandemic, telehealth usage returned to minimal utilization among patients undergoing arthroplasty. Second, our data suggest that demographics may not influence telehealth utilization, except perhaps in younger patients. Finally, new patient evaluations are least likely to use telehealth, whereas postoperative and follow-up visits are most likely to occur over telehealth although practice variability exists in our cohort.

Although payment parity exists between telehealth and in-person visits, arthroplasty care has reverted to existing practice patterns of predominantly delivering care in-person. Our data indicate that, except for 2 months in the early pandemic, most patient clinic visits across centers have been in-person. This is a pattern that has emerged across orthopedic subspecialties. Through a statewide cohort study, Chao et al. [8] analyzed telehealth utilization across surgical subspecialties during the pandemic through September 2020. Their results found neurosurgery and urology to be the highest and orthopedic surgery to be either the lowest or second lowest in converting clinic appointments to telehealth. While the present study did not focus on identifying explanations for the return to low utilization of telehealth, this finding does not seem to be driven by low patient or surgeon satisfaction, as the orthopedic literature has identified high patient and surgeon satisfaction with telehealth [[9], [10], [11]]. Moreover, other fields in medicine, such as neurology [12], have incorporated telehealth into a significant portion of their practice, with certain clinics delivering over 25% of their care through telehealth. Despite low current usage of telehealth in orthopedic surgery, a systematic review by Jenkins et al [13] forecasts growth in utilization of telehealth as technological platforms and indications for telehealth usage mature.

Among arthroplasty patients, younger patients (<50 years of age) were more likely to use telehealth, while sex does not have an impact on telehealth utilization. The results of the present study are similar to those of Xiong et al. [14], who found that across two urban academic medical centers, telehealth users tended to be younger, whereas sex did not affect telehealth utilization. Their study was from March through May 2020; the present study adds to the literature by demonstrating these patterns persist through April 2021.

Of all arthroplasty clinic visits, new patient visits were least likely to take place over telehealth. In the present study, postoperative and follow-up visits were most likely to be performed over telehealth. It is possible that patients and surgeons feel these generally lower complexity visits are more appropriate to happen via telehealth, particularly during an ongoing pandemic. It is also possible that surgeons and/or patients feel an evaluation for surgical vs nonoperative care requires an in-person physical examination. However, other authors have shown that many orthopedic surgeons feel confident in diagnoses made over telehealth [10,15]. Furthermore, in a study by Crawford et al. [16], surgical plans delineated over telehealth were compared to final surgical plans after in-person visits. Across orthopedic surgery subspecialties, it was found these plans rarely change, and in arthroplasty, in particular, the plans did not change. These results should provide patients and surgeons the assurance that any type of arthroplasty visit can likely be performed effectively over telehealth in most patients.

There are several strengths to this study. First, it is multicenter, involving multiple regions and practice types, which allows for improved generalizability. Furthermore, it is the first arthroplasty study, to our knowledge, to analyze telehealth utilization over a 1-year period. There are limitations to this study, including reliance on accurate and consistent visit coding by each individual center. Furthermore, this study did not investigate reasons for intercenter or intracenter differences in telehealth utilization patterns over time. Moreover, another limitation is the retrospective nature of the study, making it subject to bias. Finally, this study did not differentiate patients based on diagnoses or treatment types, which may be a future study direction.

In conclusion, among arthroplasty clinics, telehealth utilization peaked during March and April of 2020, the height of the early pandemic, and has since reverted to near prepandemic clinical practice levels. Younger patients of either sex are more likely to use telehealth for arthroplasty appointments. Lower complexity visits such as postoperative visits or follow-up visits, as opposed to new patient evaluations, are more likely to use telehealth. We remain in the early phases of telehealth use in health care, and improvements in technology and regulation may make this mode of health-care delivery more commonplace in the future.

## Conflicts of interest

M. Ast is in the editorial or governing board of *Journal of Arthroplasty* and is a committee member of AAHKS, AAOS, and EOA. J. D. Maratt is in the speakers’ bureau of or gave paid presentations for and is a paid consultant for Medacta; and is a board member of AAHKS Digital Health. C. A. Krueger is a paid consultant for Smit and Nephew and is a board member of AAOS and AAHKS. S. Bini received royalties from Stryker, has stock or stock options in InSilicoTrials.com, CaptureProof.com, Cloudmedix.com, and SiraMedical.com; received research support from Google.com, received financial or material support from Elsevier, is in the editorial or governing board of *Journal of Arthroplasty* and *Arthroplasty Today*; and is a board or committee member of American Association of Hip and Knee Surgeons and Personalized Arthroplasty Society.

## Figures and Tables

**Conflict of Interest Statement for Bini:**
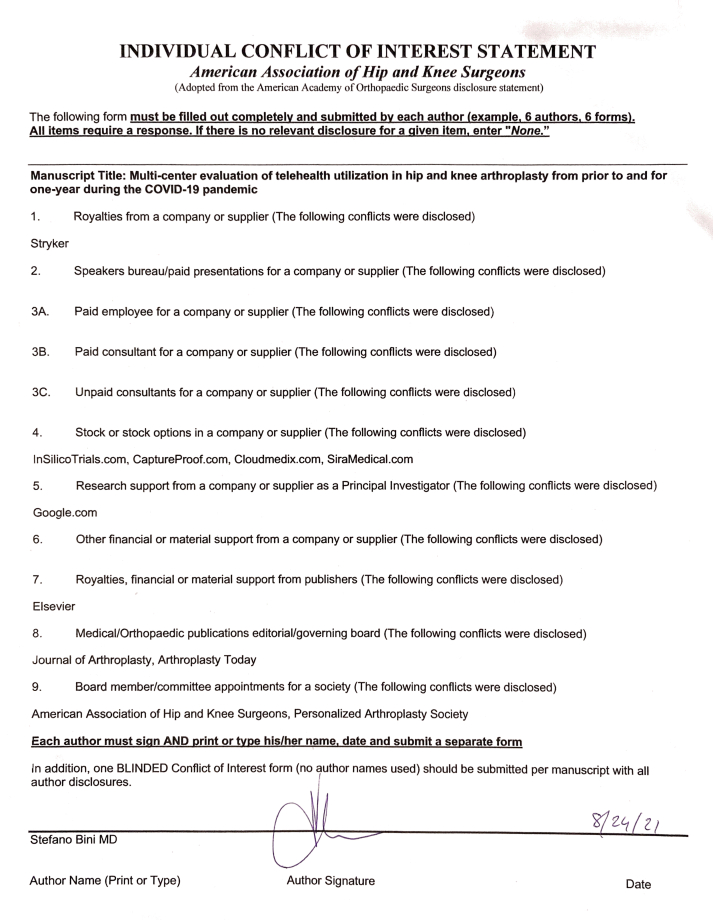


## Supplementary data

Conflict of Interest Statement for Bendich

Conflict of Interest Statement for Chiu

Conflict of Interest Statement for Maratt

Conflict of Interest Statement for Ast

Conflict of Interest Statement for Krueger
